# HPV self-testing for primary cervical cancer screening in Madagascar: VIA/VILI triage compliance in HPV-positive women

**DOI:** 10.1371/journal.pone.0220632

**Published:** 2019-08-13

**Authors:** Anne-Caroline Benski, Manuela Viviano, Jéromine Jinoro, Milena Alec, Rosa Catarino, Joséa Herniainasolo, Pierre Vassilakos, Patrick Petignat

**Affiliations:** 1 Gynecology Division, Geneva University Hospitals, Geneva, Switzerland; 2 Saint Damien Health Center, Ambanja, Madagascar; 3 University of Geneva, Geneva, Switzerland; 4 Geneva Foundation for Medical Education and Research, Geneva, Switzerland; Azienda Unita Sanitaria Locale di Reggio Emilia, ITALY

## Abstract

**Objective:**

To assess triage compliance and the effect of the time from screening to triage on follow-up among HPV-positive women.

**Methods:**

We recruited 1232 women in a screening campaign in Madagascar from February to October 2015. In the first period (February–May), HPV tests were performed remotely using the cobas test. In the second period (May–October), testing was performed on-site using the Xpert HPV assay. HPV-positive women were invited for triage with visual inspection with acetic acid (VIA) and Lugol’s iodine (VILI). Systematic biopsy and endocervical brushing were performed on all HPV-positive women for quality control. Three groups were defined according to time from HPV testing to triage invitation for HPV-positive women—Group I: delayed (> 3 months), Group II: prompt (24–48 hours), and Group III: immediate (< 24 hours).

**Results:**

A total 1232 self-sampled HPV tests were performed in the study period (496 in Group I, 512 in Group II, and 224 in Group III). Participants’ mean age was 43.2 ± 9.3 years. Mean time from screening to VIA/VILI testing was 103.5 ± 43.6 days. Overall HPV prevalence was 28.0%. HPV prevalence was 27.2% in Group I (cobas test), 29.2% in Group 2 (Xpert test), and 26,7% in Group III (Xpert test). The VIA/VILI compliance rate was 77.8% for Group I, 82.7% for Group II, and 95.0% for Group III. Of women undergoing VIA/VILI, 56.3% in Group I and 43.5% in Groups II/III had positive results. Prevalence of cervical intraepithelial neoplasia grade 2 or worse among HPV-positive women was 9.8% for Group I and 6.8% for Groups II/III. Non-adherence was higher among rural women, uneducated women, and women in Group I.

**Conclusion:**

HPV-positive women with immediate VIA/VILI triage invitation had the best triage compliance. A single-day test and triage strategy is preferred for low-resource settings.

## Introduction

Cervical cancer (CC) is the third most common cause of cancer mortality in lower-middle-income countries (LMIC) [1]. Worldwide, approximately 311,000 women died from CC in 2018, with deaths in LMIC accounting for 85% of this Fig 2. According to the World Health Organization, each year, 34 out of every 100,000 women in Africa are diagnosed with CC, and 23 out of every 100,000 women in Africa die from the disease [3]. In sub-Saharan Africa, in particular, CC is the most common cancer among women, mainly because of difficulties in implementing screening and treatment services [4,5]. CC ranks as the most frequent cancer among women in Madagascar.

Visual inspection of the cervix with acetic acid (VIA) and Lugol’s iodine (VILI) is the nationally recommended approach for CC screening in Madagascar, as is the case in most LMIC. VIA/VILI has the advantages of being low-cost and providing immediate results, allowing screening and treatment during the same visit [6]. A 2015 meta-analysis of 29 studies on VIA and 19 studies on VILI reported the sensitivity for detecting cervical intraepithelial neoplasia grade 2 or worse (CIN2+) to be 73.2% for VIA and 88.1% for VILI [7]. The same study reported the specificity of the tests to be 86.7% for VIA and 85.9% for VILI. The main weakness of VIA/VILI-based screening is a high level of dependence on individual expert care-providers whose performance varies widely, and the subjective nature of this type of screening makes quality control and quality assurance through supervision and regular monitoring especially critical [8]. Quality indicators should focus on screening rate, positivity rate, treatment rate, and coverage rate [9].

Human papillomavirus (HPV) infection is an important cause of CC. There are many subtypes of HPV, most of which do not cause CC, and a large majority (90%) of HPV infections do not persist beyond two years; however, 70% of cases of CC and precancerous cervical lesions are caused by the two highest-risk types of HPV (HPV-16 and HPV-18) [2]. The increasing availability of HPV tests makes HPV testing a feasible option for CC prevention. Such HPV-based methods improve the objectivity and performance of CC screening by detecting cases of intraepithelial neoplasia that visual approaches may fail to detect [10]. HPV detection is therefore a simple and useful screening method that has been approved by the World Health Organization as an option for primary screening, to be followed by VIA as a triage test [11].

HPV tests generally require that samples be sent to distant reference laboratories for analysis, making the same-day screen-and-treat strategy impractical. A two-stage protocol including HPV testing and VIA/VILI triage with treatment (if needed) performed on different days requires a recall system and may result in a significant drop-out rate [12]. It is a well-recognized phenomenon that, in the process of screening, triage, and treatment, each additional visit required incurs additional loss to follow-up [13,14]. Delays between screening, triage, and treatment are clinically problematic [15], and it is logical to hypothesize that longer delays are associated with increased drop out, although little work has explicitly examined this assumption.

Newly available on-site rapid HPV tests may reduce the length of time between HPV testing and invitation for VIA/VILI triage [16,17]. These tests could be used in LMIC because they are simple to use and require limited laboratory training. In addition, they do not need running water or air conditioning. Another advantage is that the tests can be performed on self-collected specimens, thus minimizing the need for human resources and obviating potential cultural barriers. A recent meta-analysis found that HPV self-testing can significantly increase CC screening uptake, although more work is needed to examine associations with follow-up testing and treatment [18]. Furthermore, several studies have found evidence that HPV self-testing is as accurate as clinician-collected specimens for the detection of high-risk HPV [19,20], as well as CC lesions [21].

In this study, our aim was to examine the compliance of HPV-positive women invited for triage with different time delays and to assess the influence of the delay period on the follow-up rate. Additionally, we aimed to identify factors that may influence adherence to triage.

## Materials and methods

### Study design

The study reported in this article was a secondary analysis of data from a larger study. The present study can be considered a cohort study nested within a randomized controlled trial. The main study aimed to evaluate whether smartphones can assist health-care workers and assess the diagnostic reliability and accuracy of cervical examination using smartphone photos for VIA, compared with conventional VIA, for women testing positive for HPV. In the course of analyzing the data collected for the main study, we realized that it would be interesting to investigate differences in triage compliance between women who were provided with immediate HPV results and those who received delayed results and consequently had delayed gynecological exams. For this secondary analysis, there was no assignment: Women participated in the screening program as part of their standard care, and whether they were in the “delayed invitation” or “prompt invitation” group was determined by real-life conditions rather than being assigned by the investigators. The authors confirm that all ongoing and related trials for this drug/intervention are registered.

The CC screening campaign took place in the Saint Damien Healthcare Centre in Ambanja, Madagascar, and in five dispensaries in the surrounding rural areas from February to October 2015. Here, CC prevention is integrated with a range of reproductive and sexual health services (family planning, antenatal care, HIV and sexually transmitted disease counselling) available at the center. Women aged 30–65 years living in Ambanja and its surroundings were invited to undergo CC screening. They were asked to collect a vaginal sample with a sterile swab (ESwab, Copan, Brescia, Italy) for HPV detection after providing signed informed consent form.

In the first period (February to May 2015), The HPV samples were analyzed in Switzerland with the cobas 4800 (Roche Grenzacherstrasse 124, 4070 Basel) test. It was necessary to wait for a colleague to take the tests to from Madagascar to Switzerland, where they were analyzed (a 1-day process). The results were then returned by email for management in Ambanja. In total, this took a minimum of 2 weeks from the administration of the test to the receipt of results, which required recalling all women to the health care facility for information about the results. HPV-positive women received a delayed invitation for a VIA/VILI triage. In the second period (May to October 2015), HPV tests were analyzed on-site with the Xpert assay (GeneXpertIV; Cepheid, Sunnyvale, CA, USA), and HPV-positive women were invited either immediately (< 24 hours) or promptly (24–48 hours) to undergo triage with VIA/VILI, which was performed by trained local gynecologists.

A lower threshold for VIA positivity than is usually recommended by the International Agency for Research on Cancer criteria [12] was applied: We considered any acetowhite lesion (faint, translucent, or dense), including those that were indeterminate or uncertain and those touching the cervical transformation zone, to be positive (or pathological) results. We considered a lesion “matching” a positive VIA result to be a positive VILI result. For women accepting VIA/VILI triage, both endocervical brushing and biopsy of the lesion were performed as quality control measures. For a random sample of cases with negative VIA/VILI results, cervical biopsy with endocervical brushing was performed at the 6 o’clock site of the transitional zone. Digital images were captured using a smartphone (Samsung Galaxy S4 or S5, Seoul, South Korea). The first image corresponded to the native cervix, the second was taken 1 minute after application of a 5% acetic acid solution, and the third was taken following application of Lugol’s iodine. Women with VIA/VILI-positive triage were offered same-day treatment with thermoablation or loop electrosurgical excision procedure. Patients with a suspicion of CC confirmed by biopsy were asked to come back for a hysterectomy (if operable). Women who were not treated but were later histologically diagnosed with a high-grade lesion (CIN2 or CIN3) were recalled to the Saint Damien Healthcare Centre for appropriate therapy.

The study took place from January 1, 2015, to October 31, 2015. The original study was registered (accepted by the ethics committee after corrections) in February 2015, before the first patient was enrolled in the study in the same month. The study was approved by the representative officer of the Malachi National Commission for the Ethics of Science and Technology in Ambanja and the Ethical Cantonal Board of Geneva, Switzerland (CER: 14–071). The authors confirm that all ongoing and related trials for this drug/intervention are registered.

### HPV testing

The cobas HPV Test uses amplification of target DNA by polymerase chain reaction and nucleic acid hybridization to detect the 14 high-risk HPV types in a single analysis. The cobas test individually identifies HPV-16 and HPV-18 and reports the other 12 high-risk types of HPV (31, 33, 35, 39, 45, 51, 52, 56, 58, 59, 66, and 68) as a combined result. The Xpert HPV assay consists of a real-time cartridge-based polymerase chain reaction that enables partial genotyping among 14 high-risk HPV types. (HPV-16, HPV-18/45, HPV31/33/35/52/58, HPV51/59, and HPV 39/56/66/68). The results are available in 1 hour. In addition to reporting whether the test was positive or negative, we also report HPV co-positive test results, which indicate that the HPV test yielded “HPV-16 and HPV-18/45,” “HPV-16 and other high-risk HPV,” or “HPV-18/45 and other high-risk HPV” as a result.

### Time to triage invitation

**Fig 1:**
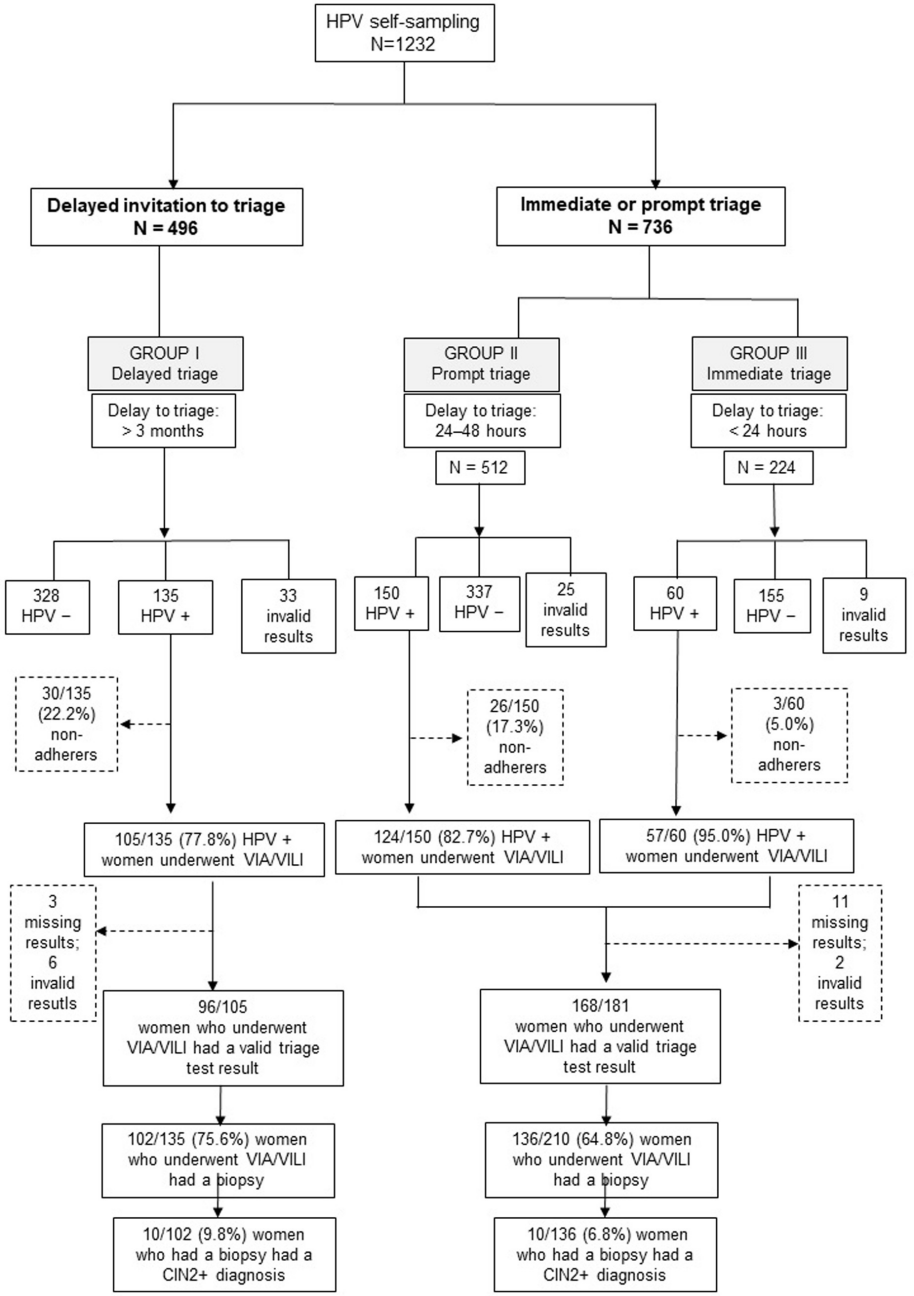
Flowchart of the study design. HPV: human papillomavirus, VIA: visual inspection with acetic acid, VILI: visual inspection with Lugol’s iodine, CIN2+: cervical intraepithelial neoplasia grade 2 or worse.

Time to triage invitation among HPV-positive women was evaluated as follows (Fig 1): Women with delayed invitation for triage (> 3 months after screening) were designated as Group I, women who were promptly invited for triage (24–48 hours after screening) were designated as Group II, and women who were invited for triage immediately after screening (< 24 hours) were designated as Group III.

### Statistical analyses

To assess the associations between the HPV test used and the participants’ socio-demographic characteristics and pathological and screening test results, the results of Fisher’s exact tests are reported when at least one of the expected frequencies was less than three; otherwise, the results of chi-square tests are reported. Compliance with VIA/VILI triage among HPV-positive women was the main outcome variable in this study. Multiple logistic regression analysis was performed including all explanatory variables with *p* < 0.20 in bivariate analyses. Statistical significance was defined as *p*-value < 0.05, and 95% confidence intervals (CI) were calculated for the results. Data were analyzed using a statistical analysis software package (StataCorp, Stata Statistical Software: Release 14, College Station, TX, USA).

## Results

### Characteristics of the study population

The study design is illustrated in Fig 1. A total of 1232 women were recruited to the study and performed an HPV self-test. Of these women, 496 received a delayed invitation for triage (Group I), and 736 received a prompt or immediate invitation for triage (Groups II and III). The participants’ mean ± standard deviation (SD) age was 43.3 ± 9.2 years in Group I and 43.0 ± 9.4 years in Groups II/III (*p* = 0.383). The mean ± SD age at first sexual intercourse was 16.6 ± 2.1 in Group I and 16.9 ± 2.1 years in Groups II/III (*p* = 0.083). Women whose highest educational achievement was elementary school made up 211/496 (42.5%) of Group I and 328/736 (44.9%) of Groups II/III. The participants’ socio-demographic characteristics are reported in Table 1. There were statistically significant differences between Group I and Groups II/III on relationship status (*p* = 0.008), employment status (*p* < 0.001), and contraceptive method used (*p* = 0.050).

**Table 1:** Sociodemographic and clinical characteristics of the study population.

Variable	Total	cobas	GeneXpert	*p*-value^a^
	N = 1232	N = 496	N = 736	
Age, mean ± SD	43.2 ± 9.3	43.5 ± 9.2	43.0 ± 9.4	0.383
Gestity, mean ± SD	5.4 ± 3.2	5.4 ± 3.2	5.4 ± 3.2	0.691
Parity, mean ± SD	4.1 ± 2.4	4.2 ± 2.5	4.1 ± 2.4	0.476
Number of sexual partners, mean ± SD	6.2 ± 5.8	6.0 ± 5.1	6.3 ± 6.2	0.406
Age at first sexual intercourse, mean ± SD	16.8 ± 2.1	16.6 ± 2.1	16.9 ± 2.1	0.083
Relationship status				
Single	377 (30.7)	131 (26.5)	246 (33.6)	0.008*
With a partner	851 (69.3)	364 (73.5)	487 (66.4)	
Education level				
None	176 (14.3)	75 (15.1)	101 (13.8)	0.572
Elementary school	539 (43.9)	211 (42.5)	328 (44.9)	
Apprenticeship	483 (39.4)	201 (40.5)	282 (38.6)	
High school	26 (2.1)	9 (1.8)	17 (2.3)	
University	3 (0.2)	−	3 (0.4)	
Employment status				
Employed	233 (19.0)	83 (16.8)	150 (20.5)	< 0.001*
Farmer	676 (55.1)	55 (11.1)	399 (54.5)	
Housewife	168 (13.8)	277 (56.1)	113 (15.4)	
Other	149 (12.2)	79 (16.0)	70 (9.6)	
Contraception				
Pill	99 (8.5)	43 (9.1)	56 (8.1)	0.050*
IUD	3 (0.3)	1 (0.2)	2 (0.3)	
Injection	222 (19.1)	96 (20.3)	126 (18.3)	
None	805 (69.2)	328 (69.2)	477 (69.2)	
Other	34 (2.9)	6 (1.3)	28 (4.1)	
Previous cervical cancer screening				
Yes	12 (1.0)	8 (1.6)	4 (0.6)	0.075
No	1209 (99.0)	480 (98.4)	729 (99.5)	
Screening context in 2015				
Rural	856 (69.5)	344 (69.4)	512 (69.6)	0.937
Urban (Ambanja)	376 (30.5)	152 (30.7)	224 (30.4)	

*p<0.05

SD: standard deviation, IUD: intrauterine device

^a^ The results of Fisher’s exact tests are reported when at least one of the expected frequencies was less than three; otherwise, the results of chi-square are reported.

### HPV prevalence, triage, and histopathology results

Fig 1 shows that the percentages of HPV-positive women in Groups I, II, and III who underwent VIA/VILI were 77.8%, 82.7%, and 95%, respectively. Among women who underwent triage with VIA/VILI, 75.6% (102/135) in Group I and 64.8% (136/210) in Groups II/III had a biopsy (*p* = 0.034). Of women who had a biopsy, 9.8% (10/102) in Group I and 7.3% (10/136) in Groups II/III received a CIN2+ diagnosis. Among participants in Group I, 96/105 who underwent VIA/VILI had valid triage test result data. A total of 3/105 women who underwent VIA/VILI had missing test result data, and 6/105 had invalid test results. Among participants in Groups II/III, 168/181 who underwent VIA/VILI had valid test result data, 11/181 had missing VIA/VILI data, and 2/181 had invalid test result data. As shown in Table 2, HPV prevalence was 28.0% (345/1232) overall, 27.2% (135/496) for Group I (cobas HPV test), 29.2% (150/512) for Group II (Xpert HPV test), and 26.7% (60/224) for Group III (Xpert HPV test) (Group I vs. Groups II/III: *p* = 0.292). There were 54/96 (56.3%) and 73/168 (43.5%) with a positive triage among women tested in the Group I and Groups II/III, respectively (*p* = 0.128).

**Table 2:** Pathological and screening test results.

Variable	Total	cobas	GeneXpert	*p*-value^a^
	N = 1232	N = 496	N = 736	
HPV test				0.292
Positive	345 (28.0)	135 (27.2)	210 (28.5)	
Negative	820 (66.6)	328 (66.1)	492 (66.9)	
Invalid	67 (5.4)	33 (6.7)	34 (4.6)	
HPV genotype				0.015*
HPV-16	60 (18.4)	16 (13.1)	44 (21.5)	
HPV-18/45	38 (11.6)	9 (7.4)	29 (14.2)	
Other high-risk HPV	229 (70.0)	97 (79.5)	132 (64.4)	
HPV co-positive test result^b^				0.088
No	284 (86.9)	111 (91.0)	173 (84.4)	
Yes	43 (13.2)	11 (9.0)	32 (15.6)	
VIA /VILI result^c^				0.128
Pathological	127 (48.1)	54/96 (56.3)	73/168 (43.5)	
Non-pathological	137 (51.9)	42/96 (43.8)	95/168 (56.5)	
Biopsy result				0.199
Negative	217 (87.5)	85/102 (83.3)	132/146 (90.4)	
CIN1	11 (4.4)	7/102 (6.9)	4/146 (2.7)	
CIN2	5 (2.0)	4/102 (3.9)	1/146 (0.7)	
CIN3	11 (4.4)	4/102 (3.9)	7/146 (4.8)	
Adenocarcinoma	4 (1.6)	2/102 (2.0)	2/146 (1.4)	
Biopsy result				0.392
< CIN2	228 (91.9)	92/102 (90.2)	136/136 (93.2)	
CIN2+	20 (8.1)	10/102 (9.8)	10/136 (7.3)	
CIN2+ women treated	12 (60.0)	4 (40.0)	8 (80.0)	0.068

*p-value < 0.05

^a^ The results of Fisher’s exact tests are reported when at least one of the expected frequencies was less than three; otherwise, the results of chi-square are reported.

^b^ HPV co-positive test result indicates that the HPV test yielded “HPV-16 and HPV-18/45,” “HPV-16 and other high-risk HPV,” or “HPV-18/45 and other high-risk HPV” as a result.

^c^ Invalid and missing results were excluded

HPV: human papillomavirus, VIA: visual Inspection with acetic acid, VILI: visual inspection with Lugol’s iodine, CIN1: cervical intraepithelial neoplasia grade 1, CIN2: cervical intraepithelial neoplasia grade 2, CIN3: cervical intraepithelial neoplasia grade 3, CIN2+: cervical intraepithelial neoplasia grade 2 or worse

### Compliance with VIA/VILI triage

**Fig 2:**
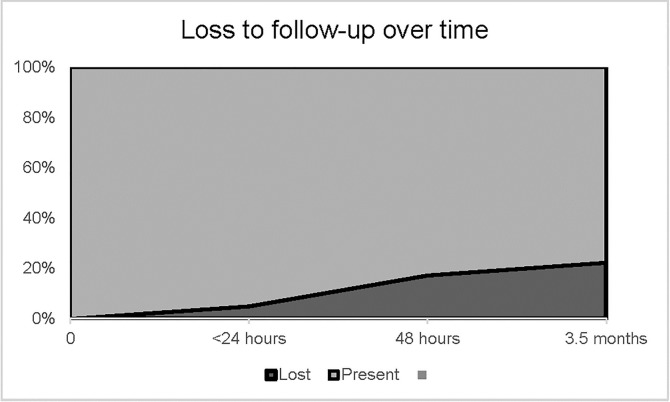
Participants lost to follow-up over time.

Overall, the mean time between screening and VIA/VILI exam was 48.4 ± 56.8 days. In Group I, the mean was 103.5 ± 43.6 days between HPV self-testing and VIA/VILI triage. The proportion of non-adherers to triage over time is illustrated in Fig 2. Of the 135 HPV-positive women tested in the Group I, 105 (77.8%) attended a VIA/VILI exam, yielding a nonadherence rate of 22.2%. In Groups II/III, 181/210 (86.2%) HPV-positive women showed up for VIA/VILI triage, representing a nonadherence rate of 13.8%. The triage adherence rate was highest when women received their HPV results on the same day as the HPV screening (Group III) (57/60, 95.0%). Adherence to triage differed significantly between Group I and Groups II/III (*p* = 0.043).

Table 3 reports the results of the crude and adjusted analyses predicting non-adherence to VIA/VILI triage among HPV-positive women, with these women’s clinical and socio-demographic characteristics included as independent variables. In the adjusted model, compared with women in Group I, the odds of failing to present for VIA/VILI triage after HPV testing were 80% lower for women in Group III (adjusted odds ratio [aOR] = 0.2, 95% CI: 0.05–0.72). The odds of not attending triage were also lower in Group II than in Group I for women in the present study, but this result was not statistically significant (aOR = 0.65, 95% CI: 0.33–1.29). Women whose highest educational attainment was high school had 63% lower odds of not attending triage, compared with women with no education (aOR = 0.37, 95% CI: 0.14–0.94). In the crude model, the odds of not attending triage for women screened in the urban setting of Ambanja were 66% lower (odds ratio = 0.34, 95% CI: 0.16–0.73) than the same odds for women screened in rural settings.

**Table 3:** Crude and adjusted analysis of factors associated with HPV-positive women not attending VIA/VILI triage.

Variable	Crude model			Adjusted model		
	OR	95% CI	*p*-value	aOR	95% CI	*p*-value
HPV test type						
cobas (Group I)	1	Reference		1	Reference	
Xpert—within 48 hours (Group II)	0.84	(0.46–1.50)	0.088	0.65	(0.33–1.29)	0.220
Xpert–immediate (Group III)	0.18	(0.05–0.63)	0.007*	0.2	(0.05–0.72)	0.014*
HPV test result**						
HPV-16	1	Reference		1	Reference	
HPV-18/45	1.92	(0.61–6.02)	0.263	0.46	(0.12–1.69)	0.240
Other high-risk HPV	1.48	(0.71–3.07)	0.298	0.63	(0.28–1.41)	0.263
Screening context						
Rural	1	Reference		−	−	−
Urban (Ambanja)	0.34	(0.16–0.73)	0.005*	−	−	−
Relationship status						
Single	1	Reference		1	Reference	
With a partner	1.06	(0.59–1.88)	0.849	1.18	(0.62–2.27)	0.61
Education level						
None	1	Reference		1	Reference	
Elementary school	0.45	(0.23–0.92)	0.027*	0.47	(0.21–1.03)	0.060
High school	0.29	(0.13–0.64)	0.002*	0.37	(0.14–0.94)	0.036*
University	−	−	−	−	−	−
Employment status						
Employed	1	Reference		1	Reference	
Unemployed	0.56	(0.18–1.71)	0.306	0.53	(0.16–1.73)	0.289
Housewife	1.66	(0.80–3.44)	0.176	0.91	(0.38–2.17)	0.837
Other	0.18	(0.02–1.43)	0.104	0.13	(0.02–1.10)	0.061
Contraception						
Pill	1	Reference		1	Reference	
IUD	−	−	−			
Injection	1.59	(0.39–6.44)	0.516	1.81	(0.42–7.72)	0.422
None	2.69	(0.79–9.21)	0.115	2.33	(0.64–8.52)	0.201
Other	1.88	(0.28–12.77)	0.519	2.88	(0.37–22.2)	0.309

*p<0.05

** Includes HPV-16 and Other HR-HPV; HPV 16 and HPV-18/45; HPV 18/45 and other HR-HPV

-Denotes missing results the numbers of cases were too small to produce valid statistical test results

HPV:human papillomavirus, VIA: visual inspection with acid acetic, VILI: visual inspection with Lugol’s iodine, OR: odds ratio, aOR: adjusted odds ratio, CI: confidence interval, IUD: intrauterine device

## Discussion

Optimal benefit of CC screening can be achieved through appropriate follow-up of abnormal test results. To be effective, the follow-up strategy must be defined by organizational strategies including an appropriate sequence of steps leading to an optimal management of women with a positive screening test[22]. Triage with VIA/VILI is simpler and cheaper than any other triage test and can be used in LMIC in the context of HPV-based screening [23]. However, screening programs that require women to return to the hospital or clinic to obtain their screening test results are associated with a high rate of non-adherence, which contributes to the reduced effectiveness of screening [12].

### Key findings and comparisons with previous work

In the present study, a prompt or, especially, immediate invitation to triage resulted in much higher VIA/VILI triage uptake when compared with a delayed invitation. This finding highlights the potential impact of a screening strategy that minimizes the number of visits on triage attendance rate. Overall, 78% of HPV-positive women had VIA/VILI triage when they received a delayed invitation (Group I), whereas 86.2% of HPV-positive women attended VIA/VILI triage when they received a prompt or immediate invitation (Groups II/III). It should be emphasized that 95% of HPV-positive women in this study attended VIA/VILI triage when their HPV test results were available immediately after the screening (Group III).

Among women in Groups II/III, supplementary analyses showed that those who received their results with a delay of less than 24 hours were four times more likely to adhere to VIA/VILI triage, compared with women who received their results from 24 to 48 hours after the screening (p = 0.005). This clearly shows the negative impact on follow-up adherence rate of even a one-day delay in providing the triage invitation. Previous studies have shown that loss to follow-up can be even greater—as high as 70%—when visits are scheduled more than 4 weeks after the initial visit [24, 25, 26]. The screening attendance among women in our Group I was slightly lower than that found in a previous study in Argentina using self-collected HPV samples analyzed with a batch model HPV test [27], but our findings were comparable to the findings of another study conducted in sub-Saharan Africa [28]. In the prompt group, our findings for screening attendance were consistent with those reported in other studies using rapid HPV testing for CC screening [29].

Other than the time interval between HPV testing and triage with visual inspection, other factors were found to be associated with non-attendance. One significant factor was living in a rural setting. This illustrates one of the biggest challenges in CC screening participation in LMIC. The distance between women’s homes and health care centers is an obstacle to screening attendance because of the difficulty of accessing transportation, organizing child care, and obtaining permission to take the necessary time off work [24,27,30,31]. Our finding of an overall HPV prevalence of 28.0% among the women in our study is in line with estimates from a large meta-analysis (24.0% for sub-Saharan Africa overall and 33.6% for the Eastern sub-region) [32].

### Strengths, limitations, and suggestions for future research

The main strength of this study is its pragmatic design and the fact that it was conducted under “real life” conditions. Our study supports the importance of a same-day strategy as a key component of efforts to reduce loss to follow-up and to achieve efficient CC screening. Because a delay of even 48 hours can impact screening uptake, efforts should be directed toward implementing a single-day test-triage-and-treat strategy in developing countries. The limitations of the present study include the fact that, although the two groups in our study population came from the same setting and were similar in terms of socio-demographic and clinical characteristics, the non-randomized design of the study limits the generalization of our results to the general population. Future studies should also consider assessing the impact-level effect of different screening methods on the rate of CC, compared with the baseline rate in the unscreened population in this context. It would also be useful to identify specific population subgroups that are unscreened or under-screened to facilitate appropriate targeted interventions.

## Conclusion

In conclusion, compliance with VIA/VILI triage among HPV-positive women is higher when the invitation to triage is issued immediately or shortly after the administration of an HPV self-test. This indicates that a single-day test-triage-and-treat strategy is preferred for LMIC. Our findings underscore the importance of point-of-care CC screening to increase screening and treatment and to decrease loss to follow-up.

## Supporting information

S1 Checklist TREND statement checklist. (PDF)

S1 Protocol Cervical cancer screening in Madagascar: Usability of mobile telemedicine for detection of precancerous lesions from smartphone photos: Study protocol. (DOCX)
