# Supplementary material for: HPV self-testing for primary cervical cancer screening in Madagascar: VIA/VILI triage compliance in HPV-positive women
Source: PLoS One. 2019 Aug 13;14(8):e0220632. doi: 10.1371/journal.pone.0220632 (PMC6692065; doi:10.1371/journal.pone.0220632)
Supplement: S1 Protocol — (DOCX) [file pone.0220632.s002.docx]

**“Cervical cancer screening in Madagascar: Usability of mobile telemedicine for detection of precancerous lesions from Smartphone photos”**

**Study Protocol**

**February 2015**

**Version 3**

**Main Investigator for “Centre Medico-Chirurgical St-Damien, Ambanja, Madagascar”:**

Dr Stefano SCARINGELLA, Director of Saint-Damien Health-Care Centre, Ambanja,

Phone +261.20.86.50.077; email: [cmc.stdamien@moov.mg](mailto:cmc.stdamien@moov.mg)

**Main Investigator for University of Geneva, Faculty of Medicine**

Patrick Petignat, Department of Gynecology and Obstetrics, University Hospitals of Geneva, phone +41.22.382.82.00; email [patrick.petignat@hcuge.ch](mailto:patrick.petignat@hcuge.ch)

**Other Investigators for University of Geneva, Faculty of Medicine**

Rosa Pinto Catarino, Department of Gynecology and Obstetrics, University Hospitals of Geneva, email [RosaIsabel.PintoCatarino@hcuge.ch](mailto:RosaIsabel.PintoCatarino@hcuge.ch)

Esther Urner, Faculty of Medicine, University of Geneva, Geneva, Switzerland, email [esther.urner@etu.unige.ch](mailto:esther.urner@etu.unige.ch)

Martine Delavy, Faculty of Medicine, University of Geneva, Geneva, Switzerland, email [martine.delavy@etu.unige.ch](mailto:martine.delavy@etu.unige.ch)

Pierre Vassilakos, Geneva Foundation for Medical Education and Research, Geneva, email [pierrevassilakos@bluewin.ch](mailto:pierrevassilakos@bluewin.ch)

**Abstract**

**Background:** Cervical cancer is the leading cause of cancer death in females in Madagascar. In this country, a large-scale screening of precancerous lesions with cytology is hardly possible, because of the lack of specialists and infrastructures. Visual inspection of the cervix with application of 5% acetic acid (VIA) is an inexpensive alternative but very subjective since it depends on the examiner’s experience. Mobile telemedicine is a very promising tool in order to assist non-expert health-care workers in rural area for cervical cancer screening.

**Objective:** To assess if Smartphone may assist health-care worker (on-site) and to evaluate the diagnostic reliability and accuracy of cervical examination with smartphone photos of VIA (D-VIA) compared with conventional VIA, for women testing positive for human papillomavirus (HPV).

**Material and method:** On-site health care workers will be trained in VIA. Prescreened HPV-positive women will be referred to VIA evaluation, during which digital images with a smartphone (D-VIA) will be taken for later evaluation by a VIA specialist in Geneva linked by telemedicine. Women with positive VIA results will be treated with cold coagulation if eligible. Histological results will be considered as gold standard. The results will be analyzed with Cohen’s kappa coefficient, Mcnemar’s test and Bonferroni’s adjustment for multiple comparisons to assess the performance of D-VIA.

**Expected results:** Based on the results of this project, we will develop an educational training and quality assurance program for health providers for VIA and so contribute to a scaling-up of cervical cancer control. An appropriate triage by VIA will reduce not only an excessive referral rate but also an excessive treatment delay, giving the possibility of a “screen (HPV), see (VIA/D-VIA), and treat” program in a single or two visits.

**Key words:** Cervical cancer; HPV; VIA; screening; smartphone; telemedicine

1. **Introduction**

Cervical cancer is the second most prevalent cancer in women Worldwide ([1](#_ENREF_1)). In Madagascar, there are about 1553 new cancer cases every year ([2](#_ENREF_2)) and, according to country statistics ([3](#_ENREF_3)), 1085 women died in 2010, which makes cervical cancer a leading cause of cancer death in females. In Western Countries, most of cervical cancer can be prevented through cytology-based or HPV screening, but for low-resource countries such as Madagascar, a large-scale screening of precancerous lesions with cytology is hardly possible, because of the lack of specialists and infrastructures.

In order to overcome barriers associated to cytology-based screening programs in low-resource countries, the World Health Organization (WHO) has recommended the use of visual inspection with acetic acid (VIA), since it is a low cost method, easy to perform and does not need specialized laboratory. Additionally, VIA allows reducing the time between when testing occurs and results are available, which allows women to be screened and treated in a single appointment (“see-and-treat approach”).

On the other side, a major drawback of VIA is subjectivity and reliability because of lack of training and expertise. The effectiveness of VIA as screening tool depends essentially from the training of the healthcare workers for diagnosis, appropriate referral and treatment of cervical pre-cancer and cancer in women living in rural Africa with limited access to care. Because the training determines the effectiveness of the VIA, it highlights the potential advantage to develop a strategy for facilitating training and supervision for novice healthcare professional working in areas where physicians are not available.

Digital imaging may offer a support for physician, but problems are that it needs digital cameras, which are expensive and require substantial field training to operate. New Smartphones appear to be an obvious choice for the development of these "next-generation" tools for digital image acquisition. Potential advantage of Smartphones is that it is “easy-use” device, allows immediate image capture and transmissions to expert via MMS in a real time for advice.

Mobile telemedicine is a very promising tool for cervical cancer screening in these countries. In view of growing applicability of this technology we will examine the feasibility, reliability and validity of a Smartphone for pre-cancer detection in low-resource countries.

1. **Objective**

Primary outcomes:

1) To evaluate if a Smartphone may assist health-care worker (in-site) for cervical cancer screening;

2) To evaluate how the expert at a distant location (off-site) may improve VIA results.

Secondary outcomes:

1) To assess the agreement between diagnoses based on the visualization of the cervix with the unaided eye, after the application of 5% acetic acid (control) during a patient clinical consultation and diagnoses for the same patient based on evaluation of Smartphone photos.

1. **Material and method**

***Inclusion Criteria:***

- 30-49 years
- HPV-positive
- Attending the cervical cancer screening program conducted by the Saint-Damien Health-Care Centre
- Understands study procedures and accepts voluntarily to participate by signing the informed consent form (ICF)

***Exclusion Criteria:***

- Previous Hysterectomy
- Conditions that can interfere with visualization of the cervix
- Pregnancy > 20 weeks
- Not able to comply with protocol study.

***Study design:***

This cohort study will be carried out in the Saint-Damien Health-Care Centre, in Ambanja, Madagascar. Women meeting the inclusion criteria will be invited to participate to the study after informed consent. Recruitment will be made during the month of July 2014.

***Study procedure:***

An informed consent form will be obtained from each woman prior to participation in the study. A questionnaire will then be filled out, assessing socio-demographic data and clinical information. These questionnaires will be available in a secured online platform containing all data from all participants. This platform will be network connected with the University Hospitals of Geneva.

A trained on-site expert will visualize the cervix with a vaginal speculum and then acetic acid 5% will be applied for at least 30 seconds. Then, a conventional VIA is performed.

In addition, acquisition of an additional set of photography of the cervix than that routinely obtained during the exam (with a digital camera or a colposcope) will be performed. The additional digital images will be obtained with a Smartphone (Samsung Galaxy S5). Photographs will be done before and after application of acetic acid and Lugol. This will result in approximately three photos per woman and should yield very little discomfort for the women.

A university-based on-site expert will independently conduct another VIA evaluation, blinded to the health worker’s evaluation.

Biopsies of the cervix (on the four forties and the lesion if any) and endocervical curettage will be performed.

All photographs will be transmitted to University Hospitals of Geneva for expert analysis. This will be made by MMS, e-mail or via RAFT network. All the images will be stored in the online platform.

The “offsite” diagnosis will be transmitted to health care worker having conducted the “in-site” diagnosis and consensus will be discussed.

The on-site health worker and the on-site university-based expert will evaluate the stored digital images (D-VIA) three months after the initial visit, and blinded to their original evaluation.

Since correlation between cytology and histology is known to be poor ([4](#_ENREF_4)), a cervical smear will be done at the end of the procedure.

Subjects deemed to be VIA positive will be treated at the same visit with cold coagulation if eligible (see flowchart).

All photographs stored in the database will be later used for educational purposes.

***Statistical analysis:***

Sample size: Based on previous studies in Ambanja (population of 28,468), Madagascar, we assume that the prevalence of CIN2+ lesions detected by D-VIA will be of 8% and of 4% by classical VIA interpretation. We have assumed 80% power at an alpha level of 0.05, using a 2-sided hypothesis. With this assumption, 1000 women will be needed.

Adequacy results: VIA and D-VIA results will be categorized as “satisfactory” (positive or negative) and “unsatisfactory”. Adequacy results (satisfactory or unsatisfactory) will be compared. Comparisons of percentages will be made between positive and negative VIA and positive and negative D-VIA. The McNemar’s test for comparisons and the Cohen’s kappa coefficient to measure reproducibility between VIA and D-VIA will be used.

Agreement results: Percentage of agreement will be calculated for each pair of on-site results and for distant D-VIA results by using McNemar’s test and adjust the p values with Bonferroni correction for multiple comparisons in order to overcorrect for Type I errors. Agreement of results will be also evaluated using Cohen’s kappa coefficient.

The following agreement results between assessors will be determined:

1) VIA results made by on-site health worker vs. distant expert D-VIA results

2) VIA results made by on-site expert vs. distant expert D-VIA results

3) VIA results made by on-site health worker vs. VIA results made by on-site expert

4) VIA results made by on-site health worker vs. on-site health worker’s D-VIA blinded results at 3 months

5) VIA results made by on-site expert vs. on-site expert D-VIA blinded results at 3 months

6) On-site health worker’s D-VIA blinded results at 3 months vs. on-site expert D-VIA blinded results at 3 months

7) On-site health worker’s D-VIA blinded results at 3 months vs. distant expert D-VIA results

8) On-site expert D-VIA blinded results at 3 months vs. distant expert D-VIA results

Accuracy: Sensitivities and specificities for the gold standard (cervical histology) will be obtained with the Smartphone diagnosis. Positive and negative predictive values will also be determined.

1. **Expected contribution**

Smartphone high-quality photographic technology has proven to be very useful in detecting pre- cancerous lesions on the cervix in a previous study that also took place in Madagascar.

Our expectation is that Teleconsultation and continuing education by mobile telemedicine will permit low-resource countries to introduce effectively and safely VIA as a triage test for those women for whom an HPV primary screening test is positive. An appropriate triage will reduce not only excessive referral rate but also an excessive delay giving the possibility of “screen (HPV), see (VIA/D-DIVA) and treat” program in a single or two visits.

**References**

1. Ferlay J, Shin HR, Bray F, Forman D, Mathers C, Parkin DM. Estimates of worldwide burden of cancer in 2008: GLOBOCAN 2008. International journal of cancer. 2010;127(12):2893-917.

2. WHO/ICO. Information Centre on HPV and Cervical Cancer (HPV Information Centre) – Human Papillomavirus and Related Cancers in Madagascar. Summary Report 2010. 2010.

3. HEALTH PROFILE MADAGASCAR. World Life Expect. At <[http://wwwworldlifeexpectancycom/country-health-profile/madagascar>.Las](http://wwwworldlifeexpectancycom/country-health-profile/madagascar%3e.Las) time accessed: 15.01.2014.

4. Marquardt K. [Correlation of cervical cytology and histology]. Der Pathologe. 2011;32(6):491-6.

1. **Annexes:**

**5.1 Flowchart**

Learning and improvement of VIA interpretation over time

(Photos used for education purposes)

**Management according to WHO guidelines**

Treat for cancer

Post-treatment Follow-up

**Treat**

Not eligible for cold coagulation

**VIA +**

eligible for cold coagulation

CANCER

CIN

Conisation

**Wait biopsy results**

**Recall**

CIN or Cancer

**VIA -**

**Wait biopsy results**

Negative

Repeat VIA in 5 years

Repeat VIA in 1 year

Negative

VIA + Biopsy

Quality control

**E-mail**

**MMS**

**HPV positive Women 30-69 years**

Followed by directed & 4-quadrant biopsies & endocervical brushing

By on-site health worker

By on-site expert

**DATA BASE (Online Platform)**

For D-VIA evaluation

Distant expert

By on-site health-worker (at 3 months)

By on-site expert (at 3 months)

**Off site diagnosis**

**On site diagnosis**

**TELECONSULTATION**

**DIAGNOSIS CONSENSUS AND MANAGEMENT**
